# Design and Expression of *Fasciola hepatica* Multiepitope Constructs Using mRNA Vaccine Technology

**DOI:** 10.3390/ijms26031190

**Published:** 2025-01-30

**Authors:** Javier Sánchez-Montejo, Tania Strilets, Raúl Manzano-Román, Julio López-Abán, Mariano A. García-Blanco, Belén Vicente, Antonio Muro

**Affiliations:** 1 Infectious and Tropical Diseases Research Group (e-INTRO), Biomedical Research Institute of Salamanca Research Centre for Tropical Diseases at the University of Salamanca (IBSAL-CIETUS), 37007 Salamanca, Spain; s.montejo@usal.es (J.S.-M.); rmanzano@usal.es (R.M.-R.); jlaban@usal.es (J.L.-A.); ama@usal.es (A.M.); 2 Infectious Department of Biochemistry and Molecular Biology, University of Texas Medical Branch, Galveston, TX 77555, USA; wvt7jr@virginia.edu; 3 Department of Microbiology, Immunology and Cancer Biology, Center for RNA Science and Medicine, University of Virginia, Charlottesville, VA 22903, USA; marianogb@virginia.edu

**Keywords:** immunoinformatics, vaccines, *Fasciola hepatica*, peptides, mRNA

## Abstract

*Fasciola hepatica* is a parasitic trematode responsible for fascioliasis, a significant zoonotic disease affecting livestock worldwide, as well as humans. This study identifies peptides with potential for use in vaccines against *Fasciola hepatica* and validates multi-epitope constructs from those peptides in vitro. Putative protein sequences derived from the genome of *F. hepatica* were integrated with phase-specific transcriptomic data to prioritize highly expressed proteins. Among these, extracellular proteins were selected using DeepLoc 2.0 and strong binding affinities across diverse human and murine alleles were predicted with the IEDB MHC II tool. Peptides were further selected based on their toxicity, immunogenicity, and allergenicity. Finally, 55 high-priority candidates were obtained. To express these candidates, mRNA constructs encoding various combinations of these peptides were designed, synthesized using in vitro transcription with T7 or SP6 RNA polymerases, and transfected into cells for expression analysis. SP6 polymerase produced proper capping using CleanCapAG and was far superior in transcribing peptide constructs. Peptides fused in frame with eGFP were expressed efficiently, particularly when peptides were positioned at the 3′ terminus, opening a new field of peptide vaccines created using mRNA technology.

## 1. Introduction

Fascioliasis, caused by the parasitic liver fluke *Fasciola hepatica*, is a significant zoonotic disease impacting livestock globally, as well as humans, particularly in South America, Africa, and Asia. The World Health Organization (WHO) estimates that more than 2.4 million people are infected worldwide, with several million more at risk [1]. The economic burden from fascioliasis in livestock, including cattle and sheep, is considerable due to reduced productivity and costs associated with treatment and control. Triclabendazole has been the most effective drug used to treat fascioliasis, killing juvenile and adult stages. Nonetheless, triclabendazole-resistant strains of *F. hepatica* have been found, which limits disease management [2]. This situation emphasizes the urgent need for alternative control strategies, such as new anti-fascioliasis drugs and/or vaccines. Developing vaccines against *F. hepatica* is particularly challenging due to the parasite’s complex life cycle and sophisticated immune-evasion mechanisms. The parasite expresses a variety of molecules that modulate the host’s immune response [3]. Due to the importance of these molecules in host−parasite interactions, they have been widely used as vaccine candidates [4,5,6,7]. Previous work using single antigens has failed to provide complete protection, suggesting that an approach that uses more than one antigen would be favored.

Small (15–20 amino acid) peptides are presented by MHC class II molecules on the surface of antigen-presenting cells, inducing T-cell responses that trigger T-cell-mediated protection [8]. Rojas-Caraballo et al. collected 269 sequences of *Fasciola hepatica* proteins and predicted three immunogenic peptides that, when tested in combination, provided partial protection against infection [9]. Currently, we have access to the complete genome sequence of *F. hepatica* [10], making it possible to apply this strategy of predicting putative protective sequences to all *Fasciola* protein-coding genes.

Messenger RNA (mRNA) vaccines have been shown to be highly effective, as highlighted by the success of mRNA vaccines against COVID-19. While limited research has been conducted on the efficacy of mRNA vaccines against helminth infections, there is potential for these vaccines to serve as powerful tools in the control of these infections [11]. Furthermore, several studies have explored the application of mRNA vaccines to other parasitic diseases, including *Toxoplasma* [12,13], *Plasmodium* [14], and *Leishmania* [15]. We believe that mRNA technology, in combination with the T-cell peptide approach described above, could be utilized to develop multi-antigen vaccines. However, the application of mRNA technology to expressing in silico-designed peptides remains largely unexplored, as there are few experimental studies on this topic [16] and most of the existing research is limited to in silico predictions.

This study describes the development of a construct consisting of a chimeric protein composed of T-cell epitopes derived from the *F. hepatica* genome. We identified the T-cell antigens using a bioinformatic workflow that evaluated transcriptional levels, location, and antigenicity. Then, to assess the feasibility of mRNA-based delivery of these epitopes, we designed and synthesized mRNA constructs encoding various combinations of the selected peptides and analyzed their expression in human cells.

## 2. Results

### 2.1. Prediction of Fasciola hepatica Peptides as Vaccine Candidates

**Figure 1:**
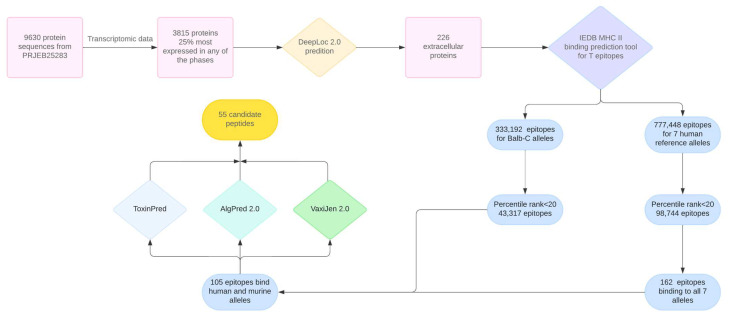
Pipeline to select 55 peptides that putatively bind seven human alleles and one murine allele of class II MHC and have high immunogenic potential against *Fasciola hepatica*.

To study which peptides would be the most suitable to elicit an immune response against *F. hepatica,* we performed bioinformatic analysis to predict the MHC-II binding peptides (Figure 1). From the 9630 protein sequences retrieved from the genome (PRJEB25283), we selected those encoded by the top 25% most-abundant mRNAs in metacercariae, newly excysted juveniles (NEJs), juveniles, and adults, according to the transcriptomic data generated by Cwiklinski et al. [17] (ERP006566). With this information, we selected 3815 proteins, which we analyzed using DeepLoc 2.0 to predict their probable localization; of these, 226 were predicted to be extracellular and, therefore, prone to contact with the host immune system. Next, we applied NetMHCIIpan-4.1 to these 226 proteins and obtained 777,448 predicted peptides that putatively bind the seven human HLA alleles and 333,192 peptides that putatively bind the murine H2 alleles. Afterward, we identified peptides predicted to have the highest affinity by using the tool, ranking the peptides and dropping those ranked below 20 for the seven selected human alleles and at least one of the murine alleles; this process yielded 105 peptides from 45 different proteins.

The 105 peptides and their characteristics are shown in Supplementary Table S1. We analyzed the physicochemical characteristics, including toxicity (ToxinPred 1.0), allergenicity (AlgPred 2.0), and antigenicity (VaxiJen 2.0), of the 105 peptides using the above bioinformatic tools, and 55 peptides were selected. These 55 peptides came from 27 proteins; seven were previously described *Fasciola hepatica* proteins, and the remaining 20 were proteins predicted from genome sequences.

### 2.2. mRNA Design and Evaluation of Multi-Peptide Constructs

**Figure 2:**
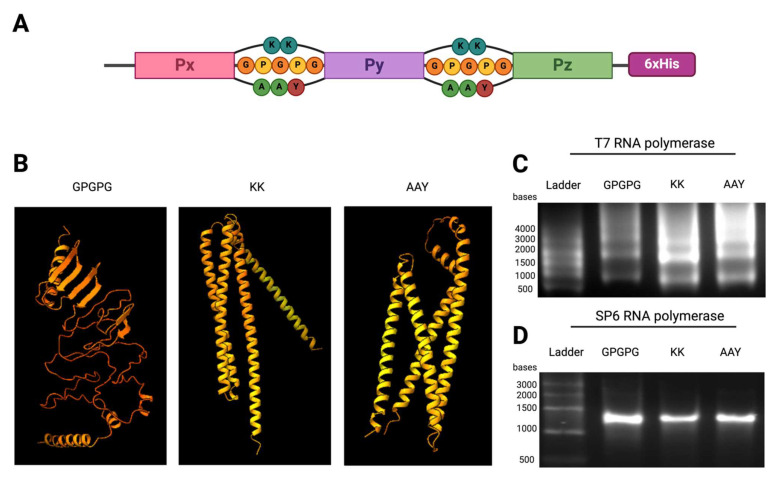
Schematic of the prediction of AlphaFold structures and in vitro transcription of multi-peptides constructs using the GPGPG, KK, and AAY linkers (**A**). Schematic representation of a construct of 3 peptides (Px, Pz, Py) fused by one of the linker combinations (GPGPG/KK/AAY) and ending in a 6xHis tag. (**B**). AlphaFold predictions of secondary structures of the 15-peptide constructs with different linkers. (**C**). T7-transcribed mRNAs from 15-peptide constructs with each combination of linkers, resolved in 2% agarose gels. (**D**). SP6-transcribed mRNAs from 15-peptide constructs with each combination of linkers, resolved in 2% agarose gel electrophoresis.

We designed and cloned into our expression vector a range of constructs comprised of a varying number (3–6–12–15–55) of the selected peptides in random order and separated by spacers composed of either GPGPG, KK, or AAY residues (Figure 2A). The constructs using the flexible linkers KK or AAY were predicted to have alpha helix structures, whereas those using linker GPGPG were predicted to be mostly disordered (Figure 2B). The mRNAs coding for our poly-antigenic proteins were synthesized using T7 IVT reactions and evaluated by agarose gel electrophoresis. Our results showed that all tested constructs encoding three or more peptides exhibited a ladder-like pattern in the agarose gel (Figure 2C). Interestingly, only the constructs with a length of three peptides were correctly transcribed, showing a single clear band (Supplementary Figure S1A). We tested modified reaction conditions to avoid cryptic stop sequences from T7 RNA polymerase, but none of the assayed conditions solved the issue (Supplementary Figure S1B).

Given the issues with synthesizing a single mRNA product using T7 RNA polymerase, we decided to change to SP6 RNA polymerase. We modified the SP6 promotor to adapt it to the capping technology and repeated IVT. For all tested constructs, the SP6 RNA polymerase produced single transcription products of the correct size, as visualized on agarose gels (Figure 2B). Therefore, we concluded that testing of multiple DNA-dependent RNA polymerases should be considered when confronting problems with IVT stalling.

Finally, we transfected the in vitro synthesized mRNAs into HEK293T cells and assayed them for protein production. Control mRNAs that encoded eGFP were produced by SP6 and capped with CleanCap AG and produced a strong fluorescence signal in cells (Supplementary Figure S2A), with subsequent detection by WB (Supplementary Figure S2B). However, no synthesized proteins were detectable by western blot in several experiments for any of the tested mRNAs encoding the multi-peptide constructs.

### 2.3. mRNA Multi-Peptide Constructs Expressed as Fusion Proteins with eGFP

**Figure 3:**
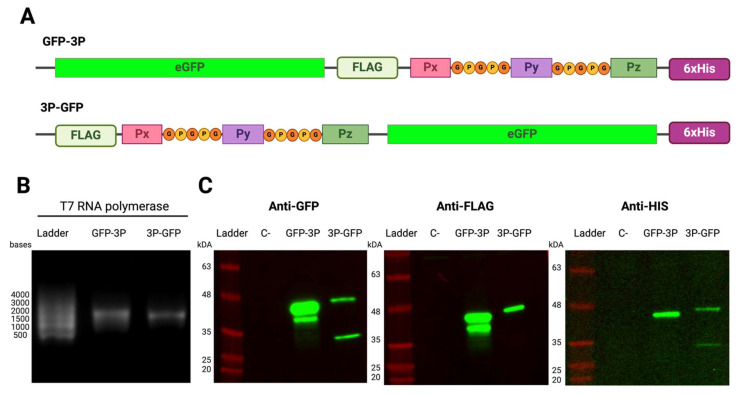
Multi-peptide constructs associated with a carrier molecule. (**A**). Schematic of the triple-peptide fusion constructs with eGFP on either the N- or C-terminus (3P-GFP and GFP-3P, respectively) (**B**). T7-transcribed mRNAs with standard reaction conditions resolved on a 2% agarose gel. (**C**). WB results of HEK293T cells 24 h post-transfection with the 3P-GFP or GFP-3P mRNA constructs. Tags were detected using an anti-GFP antibody, anti-FLAG antibody, or anti-6x histidine antibody.

We believed that fusing the chimeric peptides to a protein might help in the production of these multi-antigen chimeras. Therefore, two types of fusion proteins were designed, placing in frame three T-peptides with an eGFP protein on either the N- or C-terminus (3P-GFP and GFP-3P, respectively, in Figure 3A). Additionally, FLAG and x6His tags were introduced, flanking the antigenic peptide sequences added to either side of the constructs to help determine the effect of the presence of the peptides on the expression of the fusion protein. A single band for both N- and C-terminal eGFP-tagged constructs was observed for T7 RNA polymerase IVT (Figure 3B). Subsequently, the mRNAs were transfected into HEK293T cells and protein production was evaluated. As can be observed in Figure 3C, both constructs (3P-GFP and GFP-3P) translated completely, as evidenced by staining by the terminal histidine tag antibody (Figure 3C Anti-HIS). Fusion of the peptides to eGFP on the C-terminus (GFP-3P) produced higher protein expression than did fusion on the N-terminus (Figure 3C Anti-GFP). Furthermore, more than one band could be observed in each of the lanes, as was easily evidenced by staining with anti-GFP antibody. GFP-3P shows two bands of different sizes in both anti-GFP and anti-FLAG WB, but only one in the anti-His, suggesting that the 6xHis Tag and some of the peptides had split from the main protein. This observation can also be inferred from the 3P-GFP construct in which the tag placement is inverted, and we observed two bands in both anti-GFP and anti-HIS but only one band in anti-FLAG. These data suggest that a chimera with a well-folded protein such as EGFP may show superior expression compared to a multi-peptide protein.

## 3. Discussion

Bioinformatic predictions have become a vital tool in the initial stages of vaccine development following the reverse vaccinology approach [18]. Our study employed whole-genome analysis and an algorithm to identify peptides that could be candidates for use in a multi-antigenic vaccine, a strategy employed in the past to generate specific immune responses against parasites [9]. We began with more than 9000 putative proteins from the *F. hepatica* genome, selecting those with high transcriptional expression in all parasite life stages [17] and then predicting extracellular localization to ensure contact with the host [19]. This list was reduced to 105 peptides and refined to 55 peptides predicted to be immunogenic [20], non-toxic [21], and non-allergenic [22]. These 55 peptides came from 27 proteins, 20 of which are putative and have not been used as vaccine candidates before. Of the 7 identified proteins, cathepsin L and thioredoxin peroxidase have demonstrated partial protection against disease when used as vaccine candidates [23,24]. Additionally, thioredoxin peroxidase has been linked with the activation of Th2 macrophage responses in *F. hepatica* infection [25] and is involved in the regulation of peroxiredoxins. This protein has been used as a vaccination candidate, producing partial protection in animal models [26]. We believe that mRNA technology and the design of multi-peptide constructs can be combined to develop a multi-antigenic vaccine. Therefore, a collection of multi-peptide protein constructs was designed, cloned, and assayed in vitro to establish a method for producing this type of vaccine. Our experiments revealed a significant difference in the performance of T7 and SP6 RNA polymerases for IVT of the mRNA constructs, as none of the T7 IVT reactions produced a single clear product. Although T7 polymerase is widely used for its efficiency and processivity, some of its mechanisms still need to be better understood. One notable issue with T7 polymerase is that although its natural termination sequence is relatively inefficient [27], other cryptic stop sequences located through the mRNA can terminate its transcription. G-C-rich sequences are prone to forming hairpin structures, such as those that encode the GPGPG linkers, which have been described to terminate T7 transcription prematurely [28]. Furthermore, the repeated nature of linkers in tandem can create secondary structures that might contribute to the early termination of T7 transcription or the creation of snap-back elongation [29]. Interestingly, the KK and AAY variants, which have G-C contents much lower than that of the GPGPG variant, were not correctly transcribed with T7. These findings suggest that the issue is not related to the G-C content but may instead be due to the repeated nature of the linkers themselves. In addition, only the smallest assayed constructs produced a single band, suggesting that the number of repeated sequences may be the obstacle.

As none of the conditions solved the issue, we decided to attempt synthesis of our mRNA constructs using an alternative RNA polymerase. Both SP6 and T3 RNA polymerases are viral polymerases used to perform IVT. In the case of T3 RNA polymerase, the reaction conditions differ significantly from those needed for T7 and its use is far less common, but it could be considered an alternative for some reactions [30]. In our experiments, the SP6 polymerase successfully transcribed the constructs that T7 did not, producing clear, single-band mRNA products, as confirmed by agarose gel electrophoresis. This implies that the mechanism that hinders T7 transcription is not present in SP6 polymerase. CleanCap co-transcriptional capping is widely described as a method used with T7 RNA polymerase [31]; however, its use with SP6 RNA polymerase has been suggested but not published yet. As observed from a protein product translated and expressed by the eGFP controls used in this study, SP6-transcribed mRNAs can be properly co-transcriptionally capped by CleanCap AG (Supplementary Figure S2) using the modified SP6 promotor. However, despite successful transcription of the polypeptide’s mRNA using SP6 polymerase, none of the constructs produced detectable protein expression in HEK293T cells. The fact that the controls were properly expressed suggests that the transcription process or the capping was not the limiting factor for the expression of the designed constructs.

Our initial hypothesis was that the size of the proteins could be a limiting factor, so different, smaller constructs were tested with no different outcomes. Further insights into predicting secondary structures in the final product pointed to the protein product being unstable due to the GPGPG linkers, as proline residues tend to disrupt alpha helices and beta sheets [32,33], producing proteins without an ordered secondary structure. Therefore, those linkers were substituted for KK or AAY, which help induce the formation of an alpha helix to stabilize the secondary structure [34]. Unfortunately, the expression of none of the variants, of either size and with either linker, was detected after transfection in cells. While the lack of expression of any tested constructs does not imply that the peptides will not be effective when expressed in a suitable host, it highlights the necessity of experimental testing to verify bioinformatic predictions. In recent years, there has been a growing trend in the reverse vaccinology field wherein a sizable number of research papers rely exclusively on in silico methods to predict vaccine candidates. While these studies are valuable for initial screening, there has been growing concern within the scientific community about the lack of experimental validation to support their hypotheses [35]. As none of the tested constructs had given a detectable protein product, we theorized that the lack of detection might be due to either a lack of expression or a lack of stability of the expressed construct. Our first hypothesis involved reduced translation efficiency or instability of the final construct caused by the creation of a chimeric protein made from peptides from different proteins without a defined secondary structure. This might lead to unstable protein structures or defective ribosomal products (DRiPs) that could lead to rapid degradation or improper folding, preventing the effective accumulation of the protein in the cytoplasm and therefore preventing detection.

We adopted a common strategy used in the *E. coli* expression system to prevent the aggregation of the expressed protein and the formation of inclusion bodies. This strategy fuses the designed constructs to a properly expressed protein to help solubilize the appended protein of interest. We selected GFP as our protein of choice, as it produces a sizable amount of protein in our tested conditions and is easy to detect. We intended to clarify whether the protein was translating but then being degraded or whether it might not have been translated at all. We strategically placed tags and the peptides at different positions to distinguish between these possibilities. As the peptides with the FLAG tag were located upstream, failure to detect eGFP would have suggested that translation had not occurred. On the contrary, if GFP was expressed but the upstream FLAG tag was not detected, this result would indicate that the peptides had been translated but subsequently lost. Similarly, placing the peptides after the GFP would allow us to test whether the presence of the GFP at the 3′ end impeded the translation of the whole construct, impeded the translation of only the peptides section, or allowed for translation with subsequent loss. Our results showed that the whole protein was expressed, and the epitopes were detected in both positions using terminal tags, showing full-length expression of the constructs. However, the placement of the tags and their detection pattern on the WB were also compatible with partial degradation of the peptides after expression, indicating that even though the multi-peptide appendix was translated, it might not have been completely stable on the protein. In addition, our findings showed higher expression levels when the peptides were placed at the C-terminus, suggesting that the presence of the peptides on the N-terminus reduced the translation efficiency. This could be due to repeated regions affecting mRNA folding near the AUG proximal element [36].

These results suggest a new way of expressing multi-antigenic constructs using mRNA technology: attaching them to a carrier protein. While using a carrier protein such as eGFP may facilitate the expression of multi-antigenic constructs in mRNA, concerns arise regarding the construct’s immunogenicity, as the immune response to the carrier could mask the immune response to the target peptides. Exploring alternative carrier proteins with lower immunogenicity may be beneficial in future studies. Proteins such as human serum albumin (HSA) or other non-immunogenic scaffolds could effectively stabilize elements without eliciting strong immune responses. Alternatively, proteins with known adjuvant effects that have been used in other vaccines, such as Toll-like receptor agonists [37], plant proteins [38], and viral VLPs like HBsAg or alfalfa mosaic virus coat protein, which have been used in malaria clinical trials [39], could also be explored as alternative carrier proteins.

Furthermore, the effect of peptide constructions on the host cells should also be evaluated by toxicological analysis. Even though the in silico analyses do not anticipate any toxicity, *cytotoxicity* should be evaluated using indicators such as mitochondrial activity by MTT assay, oxidative stress, genotoxicity, or apoptosis. Nevertheless, the T-cell epitopes from the three-peptide construction were individually evaluated in previous studies by our group and did not show any toxicity [9].

Future studies will aim to analyze in depth the innate and adaptative immune response, including specific T-cell responses to the expressed peptides. Previous studies by our group showed that chemically synthesized T-cell epitopes were able to induce T-specific responses such as IL4, IL5, IL7, IL10, IL17, and IFN-γ [9].

These findings suggest that SP6 RNA polymerase is an effective alternative to T7 when dealing with difficult-to-transcribe mRNA templates. This is particularly useful after the demonstration that the co-transcriptional capping technology CleanCap AG successfully produces functional transcripts with the designed promotor and SP6 RNA polymerase. Furthermore, we have described a method that, using fusion to a stable protein such as eGFP, allows the expression of T-cell epitopes such as the vaccination candidates predicted in this study.

## 4. Materials and Methods

### 4.1. In Silico Prediction of T-Cell Epitopes

The genome data for *Fasciola hepatica* were retrieved from ParaSite (PRJEB25283), and 9630 protein sequences were extracted and crossed with the phase-specific transcriptomic data from Cwiklinski et al. [17] (ERP006566). We then filtered the proteins for the top 25% most transcriptionally active genes in any of the parasitic life stages (metacercariae, newly excited juveniles (NEJs), juveniles, and adults). Protein location was predicted using DeepLoc 2.0 [19], and only proteins classified as extracellular were selected. The remaining proteins were scanned for T-cell epitopes using the IEDB MHC II binding tool with the model NetMHCIIpan-4.1 for seven reference human alleles (HLA-DRB1*03:01, HLA-DRB1*07:01, HLA-DRB1*15:01, HLA-DRB3*01:01, HLA-DRB3*02:02, HLA-DRB4*01:01, HLA-DRB5*01:01) [40] and the two murine alleles present in BALB/c mice (H2-IAd, H2-IEd). The peptides were further filtered to select only the top 20% of peptides ranked according to their estimated binding affinity to MHC class II [41]. Subsequently, peptides that bind all seven reference human alleles and any BALB/c alleles were further screened to remove any potentially toxic or allergenic peptides that may be unsuitable for use in a vaccine. To do so, first, we used the ToxinPred [21] server with SVM-based (Support Vector Machine) prediction to remove peptides predicted to be toxic. Then, we used the hybrid model of AlgPred2 [22] with a threshold value of 0.3 to discard allergenic peptides. Lastly, we used VaxiJen v2.0 [20], with a threshold of 0.5, to discard those that would not be potentially immunogenic.

### 4.2. mRNA Construct Design and Cloning

**Figure 4:**
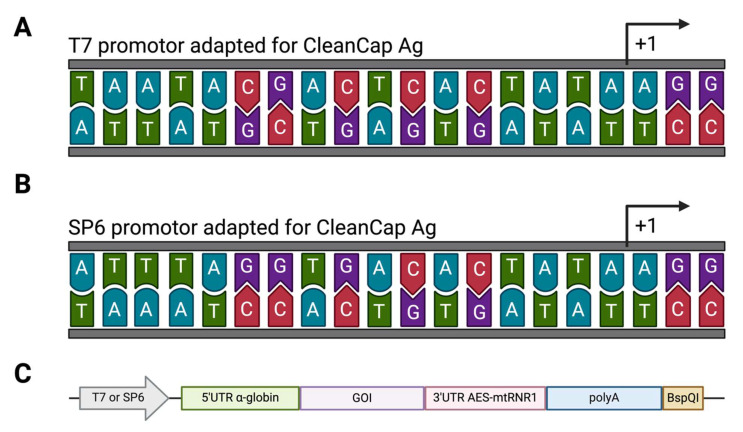
Design of the expression vector for mRNA vaccination. (**A**). Sequence of the T7 promotor adapted for use with CleanCap AG. (**B**). Sequence of the in-house SP6 promotor adapted for use with CleanCap AG. (**C**). Visual representation of the expression vector used in this study.

The expression vector was designed by placing the CleanCap AG-adapted T7 RNA polymerase promoter (Figure 4A), with the human alpha-globin 5′ UTR [42], which ensures high translational activity, in a pUC57mini backbone. This was followed by a multiple insertion site for cloning and a 3′ UTR AES-mtRNR1, which confers increased transcript stability [43]. Furthermore, a segmented 100-nt poly(A) tail interrupted by a short linker (A30LA70, where L = GCAUAUGACU) [44] followed by a BspQI restriction site was added at the end of the 3′UTR. The SP6 construct was produced by PCR using primers that included the modifications to generate an adapted version of the SP6 RNA polymerase promoter suitable for CleanCapAg (Figure 4B).

Peptides from the performed analysis were reverse translated following human and murine optimal codon usage according to GenSmart Software v1.0 (GenScript, Rijswijk, The Netherlands). Their sequences were randomly arranged in different-sized constructs and cloned in the expression vector (Figure 4C) by Gibson Assembly (Gibson Assembly^®^ Master Mix, E2611, New England Biolabs, Ipswich, MA, USA), placing the ORF after the initiation codon. All sequences were placed in the frame of a C-terminal 6xHis sequence, and, where indicated, an N-terminal FLAG-Tag sequence was also added. Secondary structures of protein constructs were predicted using the Google Colab version of AlphaFold (ColabFold v1.5.1) [45].

### 4.3. In Vitro Transcription of mRNA Constructs

The mRNA construct was produced by runoff transcription, as described in [31]. Briefly, the plasmid template for T7 constructs was linearized by digestion for 16 h using BspQI restriction enzyme (R0712S, NEB, Ipswich, MA, USA) and purified by phenol−chloroform extraction. Next, 1 µg of linearized plasmid was used as a template for in vitro transcription (IVT) using the HiScribe^®^ T7 Quick High Yield RNA Synthesis Kit yield (E2050S, NEB, Ipswich, MA, USA) with modifications. Firstly, incubation time at 37 °C was increased from 2 to 16 h. Additionally, the transcripts were capped co-transcriptionally by adding CleanCap AG (TriLink Biotechnologies, San Diego, CA, USA) to the IVT reaction to a final concentration of 4 mM. To circumvent cryptic stop sequences from T7 RNA polymerase, where specified, several reaction conditions were tested: raising or lowering the temperature of the reaction to 42 or 30 degrees, lowering or increasing incubation time to 1 h or 16 h, lowering nucleotide concentration (E2050S, NEB small fragments protocol, Ipswich, MA, USA), and decreasing the amount of template to 500 ng.

Following in vitro transcription, the linearized plasmid template was removed by incubating the crude mixture with DNAseI (M0303, NEB, Ipswich, MA, USA) for 15 min at 37 °C. Synthesized transcripts were then precipitated by adding LiCl to a final concentration of 2.5 M, incubating for 1 h at −20 °C, and centrifuging for 30 min at top speed. Then, the pellet was washed twice with 70% ethanol. The mRNAs were then resuspended in nuclease-free water and quantified using A260/A280 spectroscopy in a NanoDrop 2000 (Thermo Scientific, Waltham, MA, USA).

In cases in which SP6 polymerase was used instead of T7, the template for IVT was generated by PCR using primers that included the CleanCap AG-adapted SP6 promoter (Figure 4B). The IVT reaction was then carried out with 500 ng of PCR product using the HiScribe SP6 RNA Synthesis Kit (E2070S, NEB, Ipswich, MA, USA), following the manufacturer’s instructions and using the same conditions used for the T7 reaction. To ensure mRNA quality and integrity, IVT products were run on 2% agarose TAE gels stained with ethidium bromide and captured under UV light.

### 4.4. Expression of mRNA Constructs in HEK293T Cell Cultures

The protein production of the mRNAs was assessed by transfecting HEK293T cells using lipofectamine MessengerMax (LMRNA003, Invitrogen, Waltham, MA, USA). Briefly, 24-well plates at 70% confluence were transfected with increasing amounts (from 100 ng to 20 µg) of purified mRNA using 1 µL of MessengerMax per well, following the manufacturer’s protocol, and incubated for 24 h. To harvest, the cells were lysed in 1x RIPA buffer (9806S, Cell Signaling Technologies, Danvers MA, USA) with protease inhibitors (5871, Cell Signaling Technologies, Danvers MA, USA). Cell lysates were centrifuged at top speed for 10 min, and protein in the clarified supernatant was quantified with the Pierce BCA protein assay kit (23225, Thermo Scientific, Waltham, MA, USA). Lysates were boiled at 95 °C for 10 min in 1X SDS-PAGE Sample Loading Buffer (MB11701, NZYTech, Lisboa, Portugal) supplemented with beta-mercaptoethanol. Then, 30 µg of boiled protein in loading buffer for each sample was separated by SDS-PAGE using precast gradient gels (MB46601, NZYTech, Lisboa, Portugal) and transferred to nitrocellulose membranes (88018, Thermo Scientific, Waltham, MA, USA). Following the transference, membranes were blocked for 60 min in 5% skimmed milk. After blocking, membranes were incubated overnight at 4 °C with the following antibodies diluted, in 5% skimmed milk: 1:1000 dilution of either anti-HisTag (MA121315, Invitrogen, Waltham, MA, USA), anti-FLAG (MA1-91878, Invitrogen, Waltham, MA, USA ), anti-GFP (MA5-15256, Invitrogen, Waltham, MA, USA ), or HRP-AntiMouse-IgG secondary antibodies (A9044, Sigma-Aldritch, San Luis, MI, USA). Membranes were washed three times for 10 min each in 1x PBS + 0.1% Tween20. Membranes were incubated for 60 min with anti-mouse IgG-HRP secondary antibody (A9044, Sigma-Aldrich, San Luis, MI, USA) diluted 1:10,000 in 5% skimmed milk in PBS. After secondary antibody incubation, the membranes were once again washed three times for 10 min each in 1X PBS + 0.1% Tween20. Membranes were revealed by incubating them for 3 min with NZY Advanced ECL (MB40201, NZYTech, Lisboa, Portugal), and images were captured in a ChemiDoc (BioRad, Hercules, CA, USA).

## Data Availability

The original contributions presented in this study are included in the article/Supplementary Materials. Further inquiries can be directed to the corresponding author.

## Supplementary Materials

The following supporting information can be downloaded at: https://www.mdpi.com/article/10.3390/ijms26031190/s1.
