# Supplementary material for: Design and Expression of Fasciola hepatica Multiepitope Constructs Using mRNA Vaccine Technology
Source: Int J Mol Sci. 2025 Jan 30;26(3):1190. doi: 10.3390/ijms26031190 (PMC11818309; doi:10.3390/ijms26031190)
Supplement: Supplementary file 1 [file ijms-26-01190-s001.zip › ijms-3407116 Supplementary Figures.pdf]

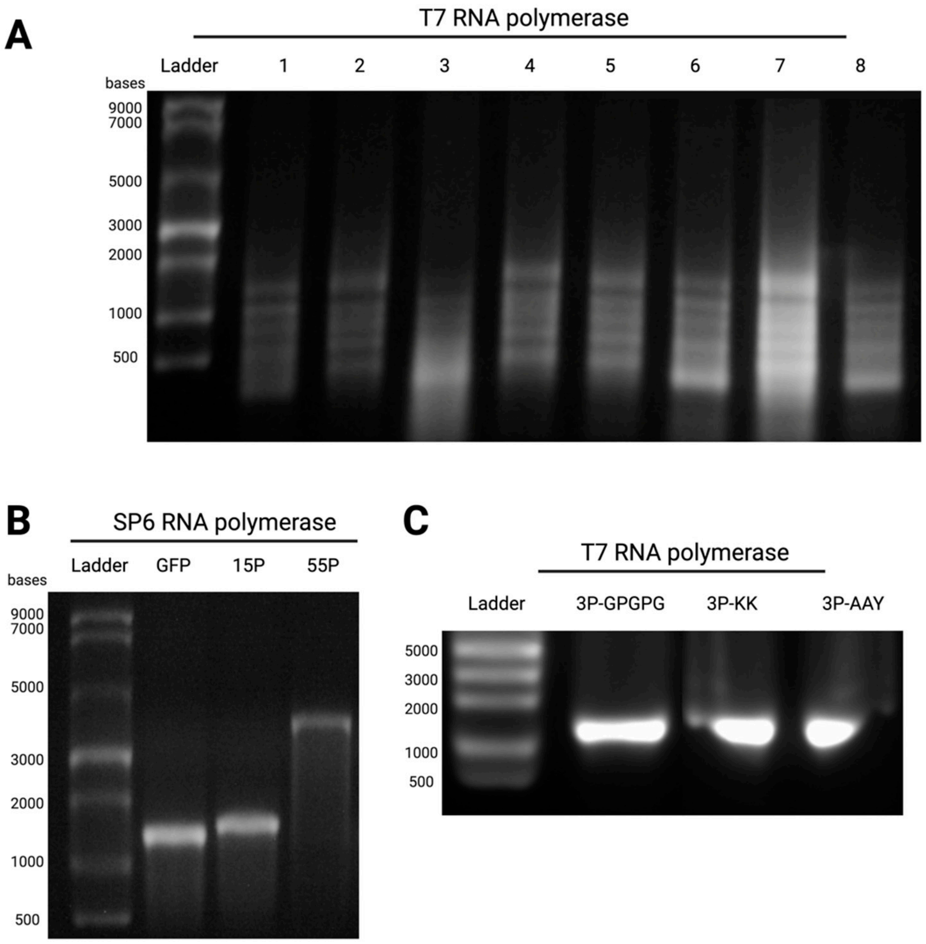

Supplementary Figure S1. mRNA transcription of multi-peptide constructs resolved in 2% agarose gel electrophoresis. A. 15-peptide constructs transcribed with T7 RNA polymerase using the following reaction conditions: 1. 30ºC 2 hours reaction; 2. 37ºC 2 hours; 3. 42ºC 2 hours; 4. 30ºC 16 hours;5. 37ºC 16 hours 500 ng template 6. 37ºC 16 hours 30 µl reaction (small fragments) 7. 37ºC 16 hours; 8. 42ºC 16 hours. B. 15- and 55-peptide constructs transcribed with SP6 RNA polymerase. C. 3-peptide constructs transcribed with T7 RNA polymerase

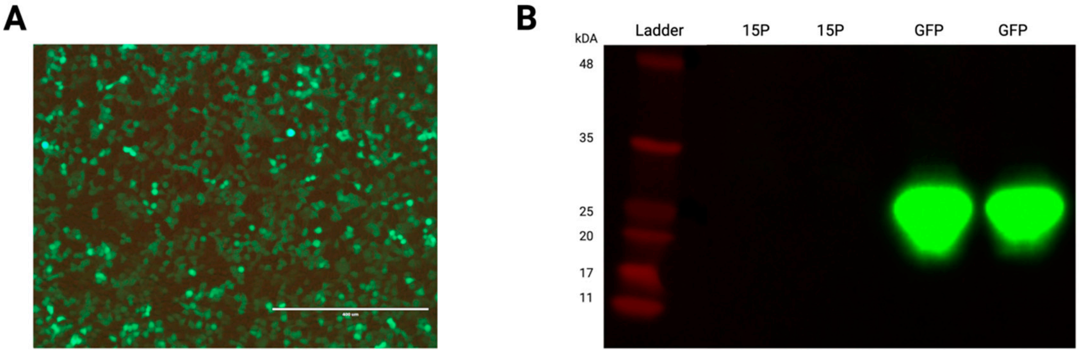

Supplementary Figure S2. Expression of SP6-produced mRNAs capped with CleanCap AG. A. Fluorescence imaging of HEK293T 24 hours post-transfection with 1 µg of CleanCap AG capped SP6 transcribed GFP mRNA. B. Anti-histidine Tag WB of 24-hour post-transfection cells with either 15-peptide construct (estimated 30 kDa protein) or GFP control.
